# Draft genome sequences of *Klebsiella* spp. isolated from produce and agricultural water in South Korea

**DOI:** 10.1128/mra.00110-24

**Published:** 2024-08-20

**Authors:** Kwang-Kyo Oh, Gyu-Sung Cho, Charles M. A. P. Franz

**Affiliations:** 1 Microbial Safety Division, National Institute of Agricultural Sciences, Rural Development Administration, Wanju, South Korea; 2 Department of Microbiology and Biotechnology, Max Rubner-Institut, Federal Research Institute of Nutrition and Food, Hermann-Weigmann-Straße, Kiel, Germany; SUNY College of Environmental Science and Forestry, Syracuse, New York, USA

**Keywords:** *Klebsiella *spp., antibiotic resistance, agricultural environment

## Abstract

This report outlines the draft genome sequences of six *Klebsiella* spp. strains from South Korea’s agricultural produce and environments. Genome sizes ranged from 5.25 to 6.21 Mbp with 55.64% to 57.55% GC content. Each strain contained multiple plasmid sequences identified by PlasmidFinder, indicating significant antimicrobial resistance.

## ANNOUNCEMENT

The genus *Klebsiella* belongs to the Gammaproteobacteria class of bacteria, of which species have been isolated from water, soil, environments, animals, and humans (1, 2). The *Klebsiella* (*K*.) spp. are well-known opportunistic pathogens that may cause infections, and these strains are also an important source of antibiotic resistance genes (3).

In this study, we report the genome sequences of six *Klebsiella* spp. samples collected from chili, Chinese cabbage, cultivation soil, and agricultural water across three provinces (Chungnam, Gyeongbuk, and Jeonnam) in South Korea. The samples were stored in sterile plastic bags, transported on ice, and processed within 4 to 6 hours. Each 25-g sample was enriched in 225 mL of nutrient broth at 37°C for 24 hours, then streaked onto MacConkey agar, and incubated at the same temperature for another 24 hours, yielding six pink, mucoid colonies. All isolates were grown in LB broth for 20 hours at 37°C under normoxic condition. Total genomic DNA was extracted using the peqGOLD Bacterial DNA extraction kit (VWR, Darmstadt, Germany) following the manufacturer’s instruction. DNA libraries were prepared using the TruSeq Nano DNA library preparation kit, and 2 × 150 bp paired-end sequencing was performed using the NextSeq 500 platform according to the manufacturers’ instructions (Illumina, Munich, Germany). The paired-end raw sequence data were trimmed using Trimmomatic (v. 0.39; parameters: Phred 33, sliding window; 4:15, leading; 3, and minlen; 36) (4), and *de novo* assembly was subsequently performed using SPAdes (v. 3.15.0; parameters: --isolate) (5). After genome assembly, contig sequences shorter than 500 bp or contaminated with the spiked PhiX genome sequence were removed using the BBDuk pipeline (BBDuk Guide - DOE Joint Genome Institute) with default parameters. The quality of the post-processed contigs was assessed using QUAST (v. 5.2.0) (6). All contigs were annotated using the NCBI Prokaryotic Genome Annotation Pipeline version (v. 6.6). For unequivocal identification of the isolates, draft genome sequences of six strains were compared to the closely related *Klebsiella* type strains, i.e., *K. pneumoniae* ATCC 13883^T^ and *K. michiganensis* DSM 25444^T^ using OrthoANI supported by USEARCH (7) and genome-to-genome distance calculator (formula 2) (8), with default parameters. Acquired antibiotic resistance genes, plasmid replicon type, and multilocus sequencing typing (MLST) were identified using the Staramr pipeline (v. 0.10.0) (9) with ResFinder (database 31.05.2023), PlasmidFinder (database 17.03.2023), and MLST (v.2.11), respectively. The genomic features of six *Klebsiella* spp. are shown in Table 1. The number of contigs ranged from 55 to 135, and the N_50_ values were between 94,183 and 189,448 for CN_Kp107 and CN_Kp115, respectively. Total genome length varied from 5.24 Mbp to 6.21 Mbp. All isolates carried more than four antibiotic resistance genes and contained at least one sequence related to plasmid replication (Table 1). The draft genome sequences of *Klebsiella* strains, isolated from agricultural water and produce, suggested the necessity for epidemiological surveillance and continuous monitoring of antibiotic-resistant bacteria in the agricultural field, particularly from a “One Health” perspective.

**TABLE 1:** Summary of whole-genome sequencing results of six *Kleblsiella* spp. strains.

	KB_Kp057	CN_Kp090	CN_Kp094	CN_Kp107	CN_Kp115	JN_Kp126
No. of contigs	69	55	58	135	59	105
N_50_	140319	172849	185818	94183	189448	114984
GC content (mol %)	57.40	57.55	57.35	57.45	57.23	55.64
Total length (bp)	5454083	5241811	5363655	5340572	5399707	6210983
Genome coverage	X47	X73	X55	X50	X50	X43
No. of raw reads	907689	1368998	1060902	933891	932446	929866
No. of CDSs	5348	5136	5278	5264	5322	6131
No. of tRNAs	46	44	49	46	46	54
No. of rRNAs	7	8	7	5	7	6
Acquired resistance gene(s)	*aadA2, blaSHV-27, dfrA12, mph(A), oqxA, oqxB, sul1,* and *sul2*	*blaSHV-81,* *dfrA1, fosA6, oqxA,* *oqxB, qnrS1,* *sul1,* and *tet(A)*	*aph(3'')-Ib, aph(6)-Id, blaLAP-2, blaSHV-27, fosA6, oqxA, oqxB, qnrS1, sul2,* and *tet(A)*	*aph(3'')-Ib, aph(6)-Id , blaTEM-1B, dfrA14 , fosA6, oqxA , oqxB, qnrS1 , sul2,* and *tet(D)*	*blaSHV-100, oqxA, oqxB,* and *tet(A)*	*aph(3'')-Ib, aph(3')-Ia, aph(6)-Id, blaOXY-1–4, dfrA21, sul1,* and *tet(B)*
Plasmid replicon type (s)	IncFIB(K) and IncFII(K)	IncFIA(pBK30683)	IncFIB(K)	IncFIB(K)(pCAV1099-114), IncN, and IncR	IncFIB(K)	IncFIA(HI1)
MLST	*Klebsiella pneumoniae 280*	*Klebsiella pneumoniae 37*	*Klebsiella pneumoniae 661*	*Klebsiella pneumoniae 1306*	*Klebsiella pneumoniae 101*	*Klebsiella oxytoca 170*
DDH	*K. pneumoniae* ATCC 13883^T^ (93.5%)	*K. pneumoniae* ATCC 13883^T^ (93.2%)	*K. pneumoniae* ATCC 13883^T^ (93.3%)	*K. pneumoniae* ATCC 13883^T^ (93.4%)	*K. pneumoniae* ATCC 13883^T^ (93.2%)	*K. michiganensis* DSM 25444^T^ (91.0%)
Average nucleotide identity (%)	*K. pneumoniae* ATCC 13,883^T^(98.97%)	*K. pneumoniae* ATCC 13,883^T^(99.0%)	*K. pneumoniae* ATCC 13,883^T^(98.98%)	*K. pneumoniae* ATCC 13,883^T^(99.19%)	*K. pneumoniae* ATCC 13883^T^ (98.91%)	*K. michiganensis* DSM 25444^T^ (98.96%)
Isolation source	Chilli	Agricultural water	Soil	Agricultural water	Soil	Chinese cabbage
SRA accession no.	SRX23442547	SRX23442548	SRX23442549	SRX23442550	SRX23442551	SRX23442552

## Data Availability

The whole-genome sequences were deposited in the DDBJ/ENA/GenBank under the accession no. PRJNA1039570. The SRA accessions are available at SRX23442547, SRX23442548, SRX23442549, SRX23442550, SRX23442551, and SRX23442552.
